# Modeling xeroderma pigmentosum associated neurological pathologies with patients-derived iPSCs

**DOI:** 10.1007/s13238-016-0244-y

**Published:** 2016-02-13

**Authors:** Lina Fu, Xiuling Xu, Ruotong Ren, Jun Wu, Weiqi Zhang, Jiping Yang, Xiaoqing Ren, Si Wang, Yang Zhao, Liang Sun, Yang Yu, Zhaoxia Wang, Ze Yang, Yun Yuan, Jie Qiao, Juan Carlos Izpisua Belmonte, Jing Qu, Guang-Hui Liu

**Affiliations:** National Laboratory of Biomacromolecules, Institute of Biophysics, Chinese Academy of Sciences, Beijing, 100101 China; FSU-CAS Innovation Institute, Foshan University, Foshan, 528000 China; State Key Laboratory of Stem Cell and Reproductive Biology, Institute of Zoology, Chinese Academy of Sciences, Beijing, 100101 China; Gene Expression Laboratory, Salk Institute for Biological Studies, 10010 North Torrey Pines Road, La Jolla, CA 92037 USA; Universidad Católica San Antonio de Murcia (UCAM) Campus de los Jerónimos, Nº 135 Guadalupe 30107, Murcia, Spain; Beijing Hospital of the Ministry of Health, Beijing, 100730 China; Department of Gynecology and Obstetrics, Peking University Third Hospital, Beijing, 100191 China; Department of Neurology, Peking University First Hospital, Beijing, 100034 China; Beijing Institute for Brain Disorders, Capital Medical University, Beijing, 100069 China; University of Chinese Academy of Sciences, Beijing, 100049 China

**Keywords:** xeroderma pigmentosum, iPSC, disease model, neural stem cell, neuron

## Abstract

Xeroderma pigmentosum (XP) is a group of genetic disorders caused by mutations of XP-associated genes, resulting in impairment of DNA repair. XP patients frequently exhibit neurological degeneration, but the underlying mechanism is unknown, in part due to lack of proper disease models. Here, we generated patient-specific induced pluripotent stem cells (iPSCs) harboring mutations in five different XP genes including *XPA*, *XPB*, *XPC*, *XPG*, and *XPV*. These iPSCs were further differentiated to neural cells, and their susceptibility to DNA damage stress was investigated. Mutation of *XPA* in either neural stem cells (NSCs) or neurons resulted in severe DNA damage repair defects, and these neural cells with mutant *XPA* were hyper-sensitive to DNA damage-induced apoptosis. Thus, XP-mutant neural cells represent valuable tools to clarify the molecular mechanisms of neurological abnormalities in the XP patients.

## INTRODUCTION

Xeroderma pigmentosum (XP) is the first discovered rare autosomal recessive genetic disorder associated with defective repair of damaged DNA (Cleaver, 1968; Epstein et al., 1970). XP is divided into eight complementation groups (XP-A to XP-G, and XP-V) that are associated with mutations in eight genes (Fassihi, 2013; De Weerd-Kastelein et al., 1972). XP patients bear molecular defects either in nucleotide excision repair (NER) or in translesion synthesis (TLS). NER is involved in the erasure of ultraviolet (UV)-induced DNA lesions or chemical-caused DNA bulky adducts (Cleaver et al., 2009). Two most common lesions induced by UV exposure are cyclobutane pyrimidine dimers (CPDs) and (6-4) pyrimidine pyrimidinone photoproducts (6-4 PPs) (Setlow and Setlow, 1962). There are approximately 30 proteins involved in the NER pathway, including XPA to XPG. Mechanistically, XPC-HR23B and CSA-CSB complexes first recognize the damaged sites in the genome and at actively transcribed genes respectively, recruit XPA to further confirm, and then XPB and XPD as two helicases unwind the DNA double strands. Subsequently the damaged sites are removed by the endonuclease XPG and XPF. Finally, the gap is repaired by DNA polymerase and DNA ligase (Scharer, 2013; Mocquet et al., 2008). In TLS, *XPV* encodes DNA polymerase η that is responsible for bypassing unrepaired lesions during DNA replication (Cleaver, 1972; Masutani et al., 1999; Chou, 2011). Dysfunction of these XP proteins results in impairment of DNA repair, leading to genomic instability and increased tumor incidence, esp. on skins (Robbins et al., 1974). Importantly, most patients in groups XPA, XPB, XPD, and XPG also exhibit progressive neurological degeneration (Grewal, 1991; Kulkarni and Wilson, 2008), characterized by microcephaly, dementia, peripheral neuropathy, and sensorineural hearing loss (Lai et al., 2013; Hayashi et al., 2004; Anttinen et al., 2008). Of note is that these neurological symptoms are most frequently observed in XPA patients (Maeda et al., 1994). While mice deficient in XPA have been created, they did not recapitulate the neurological degeneration phenotypes observed in humans (Nakane et al., 1995). The failure of mouse XPA model calls for a relevant human model system for disease mechanistic studies.

The advent of induced pluripotent stem cell (iPSC) technology has opened an unprecedented avenue to study the mechanism of rare human genetic diseases, including those caused by defects in DNA damage repair machineries (Cockayne syndrome (Andrade et al., 2012), Fanconi anemia (Liu et al., 2014), Werner syndrome (Shimamoto et al., 2014; Cheung et al., 2014)). So far, an iPSC disease model for XP is still lacking and the establishment of which will facilitate the understanding of pathogenic mechanism, i.e. in nervous system. In this study, we established a series of XP-specific iPSCs carrying different pathogenic mutations in XP genes, which were generated from XP patients’ skin fibroblasts. We observed that XPA-mutant neural stem cells (NSCs) and differentiated neurons were defective in NER and very vulnerable to DNA damage stress. Our study provides for the first time molecular clues underlying the neurodegeneration observed in XPA patients.

## RESULTS

### Generation of non-integrative iPSCs from XP patients

**Figure 1:**
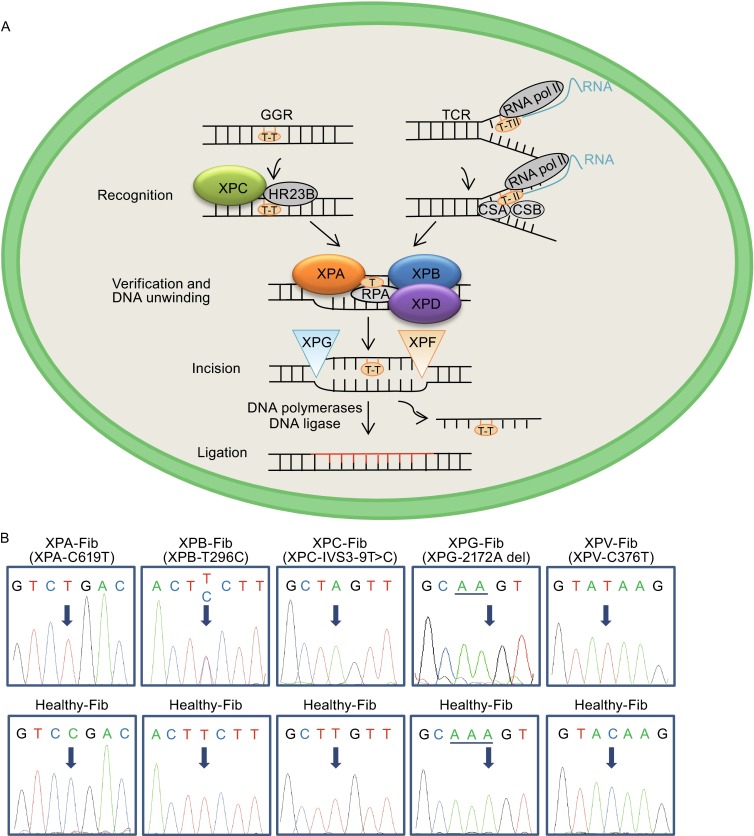
**Genotyping fibroblasts from five XP patients**. (A) Schematic diagram of NER pathway in the nucleus. Upon DNA damage, XPC and HR23B recognize damage site, XPA verifies, XPB and XPD unwind DNA double strands, finally XPF and XPG excise the damaged strand. All of these proteins function together to repair UV-induced DNA damage. GGR: Global genome repair; TCR: Transcription-coupled repair.  (B) DNA sequencing showing the different mutations in *XPA*, *XPB*, *XPC*, *XPG*, *XPV* genes in fibroblasts from five XP patients. Fibroblasts isolated from a healthy individual were used as a control

**Figure 2:**
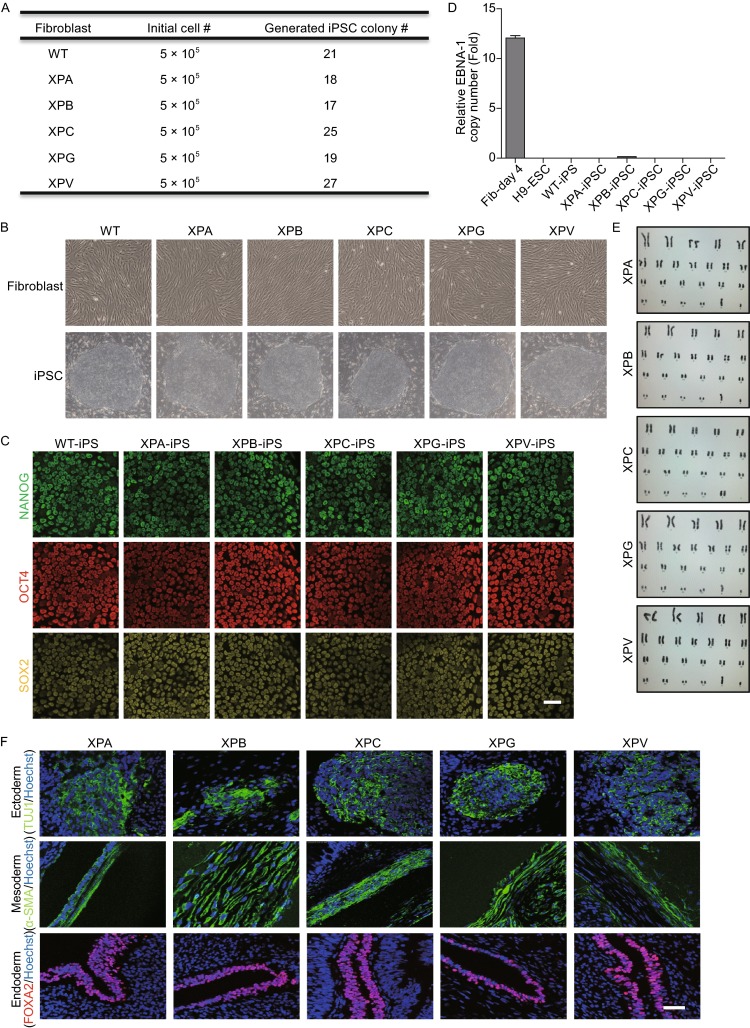
**Generation and characterization of transgene-free XP-iPSCs**. (A) Summary of the generated iPSC colony number after reprogramming of WT and XP mutant fibroblasts. (B) Phase-contrast images showing morphologies of WT, XPA, XPB, XPC, XPG, XPV mutant patient fibroblasts (top panels) and fibroblast-derived iPSCs (bottom panels). (C) Immunostaining of WT and XP-iPSCs for NANOG, OCT4, and SOX2. Scale bar, 50 μm. (D) qPCR analysis showing no or little residual episomal vector element EBNA-1 in XP-iPSCs and WT-iPSCs. Human fibroblasts 4 days after electroporated with pCXLE-hOCT3/4-shp53-F, pCXLE-hSK, and pCXLE-hUL were included as positive control, and human H9 ESCs were used as negative control. Data are shown as mean ± SD. *n* = 3. (E) Karyotyping analysis of XP-iPSCs. (F) Immunostaining for TUJ1 (ectoderm), α-SMA (mesoderm), and FOXA2 (endoderm) of teratomas derived from XP-iPSCs. Nuclei were stained with Hoechst 33342. Scale bar, 50 μm

We obtained 5 lines of human primary fibroblasts from XPA, XPB, XPC, XPG, and XPV patients, respectively. DNA sequencing analysis verified the presence of mutations in corresponding XP genes (Fig. 1A and 1B). To generate patients-specific iPSCs, we electroporated integration-free episomal vectors expressing reprogramming factors *Oct4*, *Sox2*, *Klf4*, *L*-*myc*, *Lin28*, and sh-*p53* into fibroblasts and induced them back to pluripotent state (Okita et al., 2011). All these XP gene-mutant fibroblasts were capable of being efficiently reprogrammed to iPSCs (Fig. 2A–F), despite the fact that XPC was reported as an Oct4/Sox2 coactivator by forming a protein complex in embryonic stem cells (Cattoglio et al., 2015). In addition, an iPSC line reprogrammed from healthy (WT) human fibroblasts was used as a control (Ding et al., 2015). All the derived iPSCs exhibited normal karyotype and expressed comparable levels of the pluripotency markers including NANOG, OCT4, and SOX2 (Fig. 2C and 2E). We did not detect any residual episomal reprogramming vectors in these iPSC lines (Fig. 2D). Upon being implanted subcutaneously into immunocompromised mice, these iPSCs formed teratomas comprised of cells from three germ lineages (Fig. 2F). Together, these results indicated that despite XPA, XPB, XPC, XPG, and XPV’s roles in safeguarding genome stability, mutations in these genes did not compromise somatic cellular reprogramming as well as pluripotency of generated iPSCs (Fig. 6).

### XPA-mutant NSCs demonstrated impaired DNA repair potential and increased susceptibility to apoptosis

**Figure 3:**
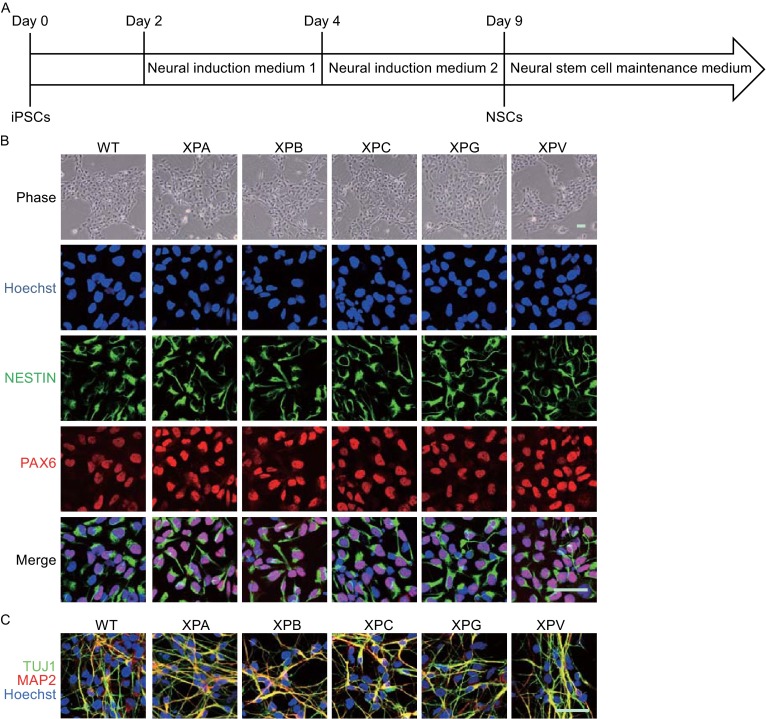
**Generation and characterization of XP-NSCs**. (A) Schematic illustration of neural stem cell differentiation from iPSCs. (B) Immunostaining of WT and XP-mutant NSCs for NESTIN and PAX6. Nuclei were stained with Hoechst 33342. Scale bar, 50 μm. (C) Immunostaining of WT and XP-mutant neurons for TUJ1 and MAP2. Nuclei were stained with Hoechst 33342. Scale bar, 50 μm

XP patients frequently exhibit symptoms of neurodegeneration, likely due to increased DNA damage in neural cells. We firstly differentiated these iPSCs into NSCs (Fig. 3A and 3B). Both WT and XP mutant NSCs exhibited typical neural progenitor morphology, expressed neural stem cell-specific markers NESTIN and PAX6 (Fig. 3B), and could be further differentiated into neurons expressing TUJ1 and microtubule-associated protein 2 (MAP2) (Fig. 3C).

**Figure 4:**
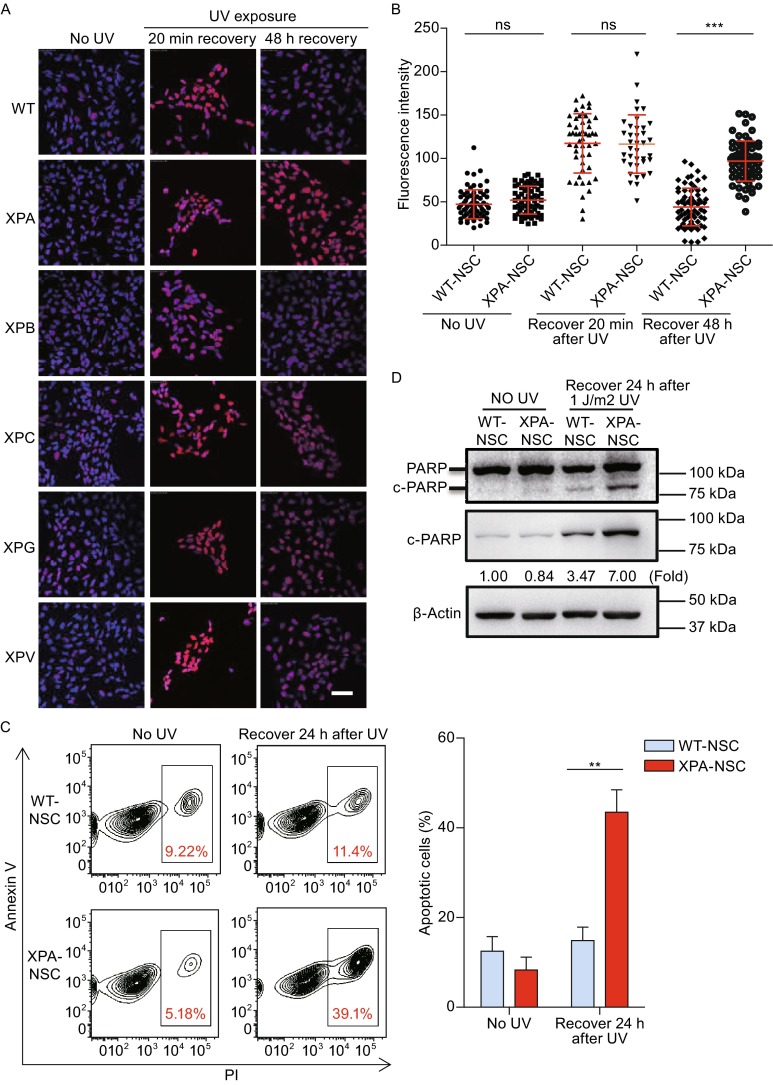

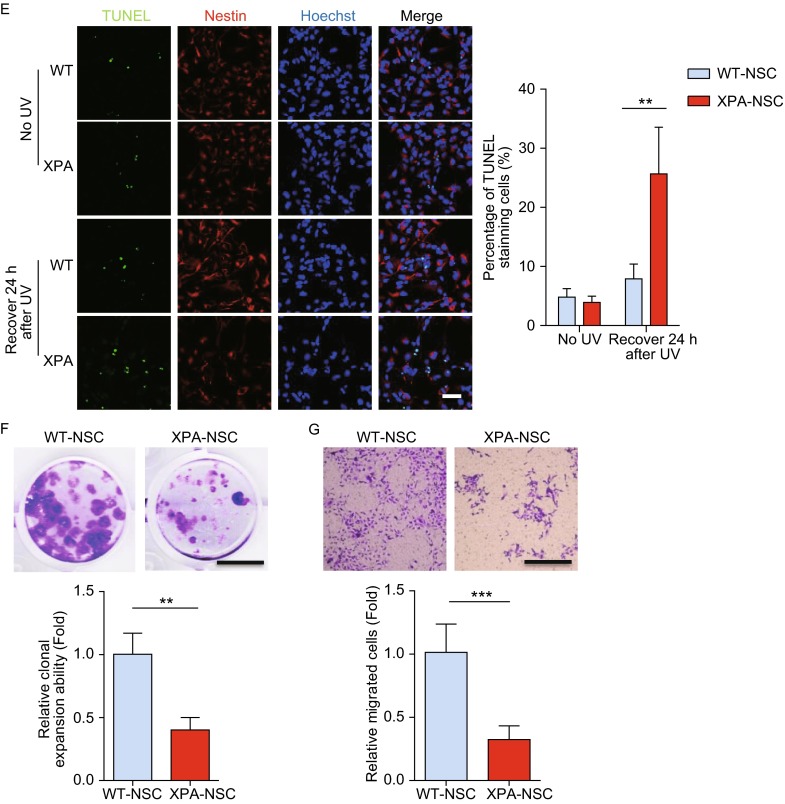
**XPA-mutant NSCs showed profound impairment of NER and hypersensitive to UV-induced apoptosis**. (A) Immunostaining of CPD in WT and XP-mutant NSCs cultured on coverslips in 24-well plate in the absence or presence of 1 J/m^2^ UV exposure. The images were taken 20 min or 48 h after UV irradiation, respectively. Nuclei were stained with Hoechst 33342. Scale bar, 50 μm. (B) Quantitative analysis of fluorescence intensity of CPD in WT and XPA-mutant NSCs in the indicated contexts. 60–100 nuclei were used for calculation. (C) Apoptosis analysis in WT and XPA-mutant NSCs 24 h after 1 J/m2 UV treatment (quantitative data shown on the right). (D) Western blots showing PARP cleavage using anti-PARP (top blots) and anti-cleaved PARP (middle blots) antibodies respectively in WT and XPA-mutant NSC 24 h after being exposed to 1 J/m^2^ UV. β-Actin was used as a loading control. (E) Representative TUNEL staining in WT and XPA-mutant NSCs cultured on coverslips in 24-well plate in the absence or presence of UV treatment (quantitative data shown on the right). Nuclei were stained with Hoechst 33342. Scale bar, 50 μm. Data are shown as mean ± SD. *n* = 3. For (C) and (D), cells were cultured on 6-well plates without coverslips. (F) Clonal expansion analysis of WT and XPA mutant NSCs. Data are shown as mean ± SD. *n* = 3. (G) Cell migration analysis of WT and XPA mutant NSCs. Data are shown as mean ± SD. *n* = 3

Next, we investigated whether mutations in XP genes could affect DNA repair ability in NSCs. UV radiation causes DNA damage by introducing CPDs and 6-4 PPs into the genome, which can be repaired via NER or TLS. Considering that normal human cells are able to repair 50% of CPDs within 24 h after UV irradiation (Nakagawa et al., 1998), we decided to employ CPD as an indicator for cellular repair activity. To this end, we challenged XP-mutant and WT-NSCs with 1 J/m^2^ UV, and determined the cellular CPD levels. Both WT and XP-mutant NSCs showed low levels of CPD at the rested state, which became strongly upregulated 20 min after UV treatment (Fig. 4A). WT-NSCs demonstrated a strong self-repair activity as the CPD dropped to basal levels 48 h after UV irradiation. In contrast, XP-mutant NSCs showed more CPD-positive cells compared to WT cells 48 h after treatment (Fig. 4A). Of note is that XPA-mutant NSCs exhibited an unusual high level of CPD 48 h after UV treatment (Fig. 4B). These observations indicated that mutations of XP genes resulted in compromised NER or TLS abilities, and XPA mutation led to most severe DNA repair defects in NSCs.

We next investigated whether compromised DNA damage repair in XPA mutant NSCs could be associated with decreased cellular survival. To this end, we treated cells with 1 J/m^2^ UV, and cellular apoptosis was determined after 24 h. We found that UV radiation resulted in massive cellular apoptosis indicated by Annexin V/PI staining in XPA-mutant NSCs while had little impact on WT-NSCs (Fig. 4C). Western blotting analysis showed increased levels of cleaved PARP (c-PARP), an apoptosis marker, in XPA mutant NSCs upon UV treatment (Fig. 4D). Additionally, terminal deoxynucleotidyl transferase (TdT)-mediated dUTP nick-end labeling (TUNEL) assay revealed more dramatic nuclear DNA fragmentation in XPA mutant NSCs following UV treatment (Fig. 4E). Additionally, we also observed that the XPA mutant NSCs had impaired abilities of clonal expansion and migration even in absence of UV radiation (Fig. 4F and 4G).

Altogether, these findings indicated a defective NER system as well as decreased cellular migration and clonal expansion in XPA-mutant NSCs, and under DNA damage stress these cells were prone to apoptosis.

### XPA-mutant neurons are defective in NER and susceptible to UV-induced apoptosis

**Figure 5:**
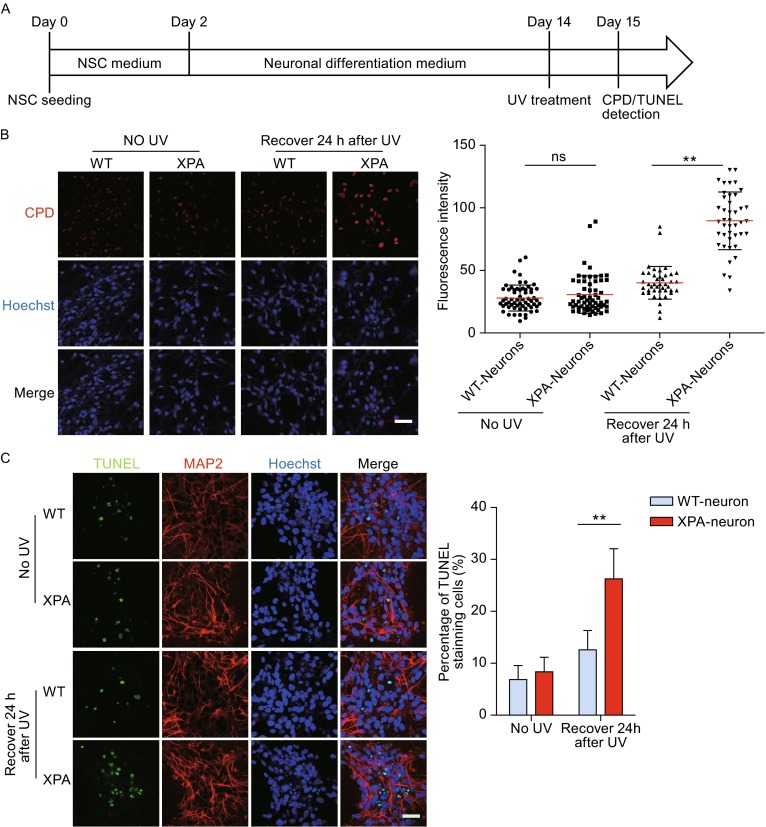
**XPA-iPSCs derived neurons showed increased susceptibility to UV-induced cell apoptosis**. (A) Schematic illustration of neuronal differentiation from NSCs. (B) CPD immunostaining in WT and XPA-mutant neurons cultured on coverslips in 24-well plate in the absence or presence of UV exposure. Nuclei were stained with Hoechst 33342. Scale bar, 50 μm. 60–100 nuclei were used for calculation. (C) Representative images of TUNEL staining in WT and XPA-mutant neurons cultured on coverslips in 24-well plate in the absence or presence of UV exposure. Nuclei were stained with Hoechst 33342. Scale bar, 50 μm. Data are shown as mean ± SD. 60–100 nuclei were used for calculation

**Figure 6:**
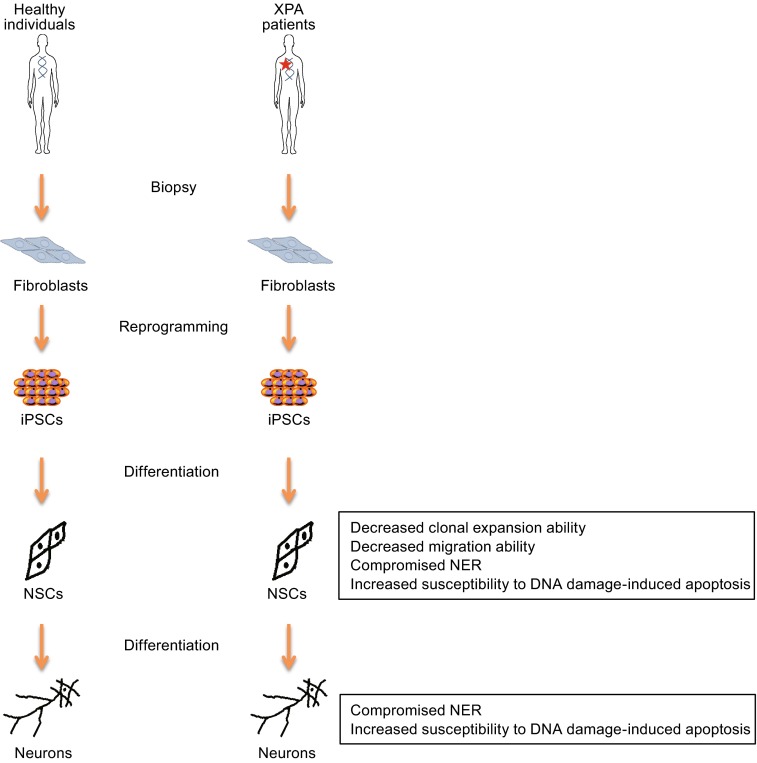
**Schematic illustration of disease modeling using XPA patient-specific iPSCs**. Both NSCs and neurons derived from XPA patient iPSCs showed compromised NER and increased susceptibility to DNA damage-induced apoptosis, which may contribute to XP-associated neurological disorders

We next investigated whether post-mitotic neurons with XPA mutation also exhibited similar defective DNA repair phenotypes. For this purpose, we differentiated WT and XPA mutant NSCs into neurons, respectively (Fig. 5A). Similar to NSCs, mutation of XPA in neurons resulted in a compromised DNA repair ability, indicated by significantly higher CPD levels upon UV treatment (Fig. 5B). Consistently, XPA-mutant neurons exhibited more TUNEL-positive cells than their WT counterparts (Fig. 5C). These results indicated XPA-mutation compromised neuron’s ability to repair DNA damage triggered by UV irradiation, and as a result apoptosis occured in XPA-mutant neurons (Fig. 6).

## DISCUSSION

Neurological abnormalities are observed in patients from certain xeroderma pigmentosum complementation groups including XPA, XPB, XPD, and XPG. However, the underlying pathogenesis basis is unclear. A roadblock to the mechanistic understanding in part can be attributed to the inability of existing XP mouse models to replicate the neurological pathologies of XP patients (Nakane et al., 1995; Andressoo et al., 2009). Therefore, a more relevant model system recapitulating human XP pathogenesis is needed for studying the disease mechanism and developing related therapy.

Here, we generated for the first time transgene-free iPSCs from fibroblasts of five XP patients bearing mutations in *XPA*, *XPB*, *XPC*, *XPG*, and *XPV* genes, respectively. While mutations of DNA repair-related genes (i.e. WRN (Shimamoto et al., 2014; Cheung et al., 2014) and Fanconi genes (Raya et al., 2009; Muller et al., 2012; Yung et al., 2013)) have been revealed as a barrier to somatic cell reprogramming, we did not observe obvious impairment in reprogramming kinetics from XP patient fibroblasts. Additionally, it has recently been reported that mutations of Fanconi genes compromised the teratoma formation efficiency (Yung et al., 2013), and deficiency of *FANCA* gene in NSCs resulted in defective neuronal differentiation ability (Liu et al., 2014). In our study, these XP gene mutant iPSCs still retained potentials to differentiate to three germ layers, including NSCs and neuronal cells. In line with these observations, XP gene mutations did not markedly affect the chromosomal stability, and the basal CPD levels are comparable between XP cells and WT cells.

Regarding the novel disease phenotypes in neural cells, we observed that mutations of XPA in patients lead to the most severe defects in NER, compared to the cells with other XP mutations. This is consistent with the observation that the patients with XPA mutation(s) more frequently exhibited neurological abnormalities. In the clinic, XPA patients showed mild to severe neuronal loss in various brain regions, including cerebral cortex, brainstem, and spinal cord (Hayashi et al., 2004). We found that the compromised NER activity not only influences the survival of the non-dividing neurons but also promotes apoptosis of dividing neural stem cells. Therefore, the neurodegenerative phenotypes observed in XPA patients may be attributed to decreased cellular survival in both NSCs and neurons upon DNA damage, as accelerated apoptosis in either NSCs or neurons could contribute to diminished number or dysfunction of mature neuronal cells, leading to neurodegeneration.

Besides neurological defects, the XP patients also demonstrated UV-associated dysfunction of skin cells, i.e. increased incidence in skin cancers. Thus the skin cell derivatives derived from XP patient iPSCs can potentially be used as a platform to study the mechanism of UV-associated malignant cell transformation. Moreover, combined with targeted gene editing techniques (Liu et al., 2011b; Suzuki et al., 2014), iPSC-derived neural or skin cells free of pathogenic mutations can also be used for cell-replacement therapies via autologous transplantation. On the other hand, the disease models established in this study hold the potential for screening candidate drugs for combating XP diseases.

## MATERIALS AND METHODS

### Cells

Human XP patient fibroblasts were purchased from Coriell Cell Repository. XPA fibroblast (GM00710) carries a homozygous 619C>T mutation resulting in a nonsense codon in exon 5 of *XPA* gene. XPB fibroblast (GM13026) is heterozygous for T>C transversion in the *XPB* gene which results in a phenylalanine-99-to-serine missense mutation. XPC fibroblast (GM15709) has a homozygous -9T>A mutation in intron 3 of the *XPC* gene. This mutation is located in a splice lariat branch point sequence. PCR analysis of fibroblast cells detected an XPC mRNA isoform with deletion of exon 4 (Khan et al., 2004). XPG fibroblast (GM13371) is identified with a 1 bp deletion in an AAA triplet at nucleotides 2170_2172 of the *XPG* gene resulting in subsequent frame shift. XPV fibroblast (GM03055) also has a nonsense mutation at nucleotide 376 of the *XPV* gene (376C>T). The control fibroblast GM00038 was purchased from Coriell Cell Repository. All these mutations in primary fibroblasts or derived iPSCs were verified by DNA sequencing. All fibroblasts were maintained in high glucose DMEM (Invitrogen) containing 15% FBS (Hyclone), 1% Glutamax (Invitrogen), 1% non-essential amino acids (Invitrogen), 1% penicillin/streptomycin (Invitrogen).

### iPSCs generation and culture

XP patients specific iPSCs were generated by electroporation of fibroblasts with episomal vectors including pCXLE-hOCT3/4-shp53-F, pCXLE-hSK and pCXLE-hUL as described (Liu et al., 2014; Okita et al., 2011; Xu et al., 2014). All the derived iPSC lines were maintained on mitomycin C-treated MEF feeder cells in hESC medium (Liu et al. 2011a, b, 2012; Zhang et al., 2015).

### Generation and characterization of NSC

NSC differentiation and characterization were performed as previously described (Duan et al., 2015; Liu et al., 2012, 2014).

### Neuronal differentiation

2.5 × 10^4^ NSCs were seeded on a Matrigel-coated well of 6-well plates. The next day, the culture medium was changed to differentiation medium containing DMEM/F12, 1× N2, 1× B27, 200 μmol/L ascorbic acid (Sigma), 400 μmol/L dbcAMP (Sigma), 10 ng/mL GDNF (Peprotech) and 10 ng/mL of BDNF (Peprotech). Laminin was added on the third day to promote differentiation. After 14 days of differentiation the neurons were subjected to characterization or cytotoxicity analysis.

### Immunofluorescence microscopy

Cells were fixed with 4% paraformaldehyde for 30 min at room temperature, washed with PBS, permeabilized in 0.4% Trion X-100 in PBS, and then blocked in 10% donkey serum (Jackson ImmunoResearch Labs). After that, cells were incubated with primary antibodies in blocking solution at 4°C overnight, followed by incubation with corresponding secondary antibodies and Hoechst 33342 for 1 h at room temperature. The primary antibodies used include anti-NANOG (Abcam, 21624), anti-OCT3/4 (Santa Cruz,5279), anti-SOX2 (Santa Cruz,17320), anti-NESTIN (Millipore, MAB5326), anti-PAX6 (Covance, PRB-278P), anti-TUJ1 (Sigma, T2220), anti-MAP2 (Sigma, 4403), anti-FOXA2 (CST, 8186), anti-α-SMA (Sigma, A5228).

### CPD immunostaining

Cells were fixed with 4% paraformaldehyde for 15 min at room temperature, washed, and then permeabilized in 0.4% Trion X-100 in PBS. Following denature of cellular DNA with 2 mol/L HCl, cells were blocked in 10% donkey serum, incubated with CPD antibody (Cosmo Bio, TMD-2) and corresponding secondary antibodies. Nuclei were stained with Hoechst 33342.

### Teratoma formation and immunohistological analyses

Five million iPSCs were injected subcutaneously into NOD-SCID mice. After 8–12 weeks, teratomas were excised, fixed, dehydrated, embedded in O.C.T compound, sectioned, and analyzed by immunostaining. All murine experiments were conducted in compliance with animal protocols approved by the Chinese Academy of Science Institutional Animal Care and Use Committee.

### TUNEL

TUNEL analysis was performed using DeadEnd™ Fluorometric TUNEL System kit (Promega). In short, cells were fixed with 4% paraformaldehyde at room temperature, permeabilized in 0.2% Trion X-100 in PBS and pre-equilibrated with equilibration buffer, followed by labeling DNA strand breaks with nucleotide mix (60 min at 37°C). 2× SSC was used to stop the reaction. DNA was stained with Hoechst 33342.

### Apoptosis

FACS-based cell apoptosis analysis was performed as previously described (Liu et al., 2012).

### Western blot analysis

For detecting the cleaved PARP in NSCs, cells were treated with UV and then protein lysates were subjected to SDS-PAGE. Primary antibodies used were anti-PARP (CST, 9542), anti-cleaved PARP (CST, 9541), anti-β-actin (Santa Cruz, 130301).

### Statistical analysis

Data are presented as mean ± SD. The statistical significance of difference between groups was calculated using Student’s *t* test. *P* > 0.05 (ns), *P* < 0.05 (*), *P* < 0.01 (**), and *P* < 0.001 (***).
